# Informing About the Nocebo Effect Affects Patients’ Need for Information About Antidepressants—An Experimental Online Study

**DOI:** 10.3389/fpsyt.2021.587122

**Published:** 2021-04-27

**Authors:** Yvonne Nestoriuc, Yiqi Pan, Timm Kinitz, Ella Weik, Meike C. Shedden-Mora

**Affiliations:** ^1^Clinical Psychology, Helmut-Schmidt-University/University of the Federal Armed Forces Hamburg, Hamburg, Germany; ^2^Systems Neuroscience, University Medical Center Hamburg-Eppendorf, Hamburg, Germany; ^3^Psychosomatic Medicine and Psychotherapy, University Medical Center Hamburg-Eppendorf, Hamburg, Germany; ^4^Neuropsychology, Westerwald Clinic, Waldbreitbach, Germany; ^5^Department of Psychiatry, British Columbia Mental Health and Addictions Research Institute, University of British Columbia, Vancouver, BC, Canada; ^6^Department of Psychology, Medical School Hamburg, Hamburg, Germany

**Keywords:** informed consent, antidepressants, nocebo effects, ethics, shared decision making, expectation, adverse (side) effects

## Abstract

**Relevance:** Understanding patients’ informational needs and adapting drug-related information are the prerequisites for a contextualized informed consent. Current information practices might rather harm by inducing nocebo effects.

**Objective:** To investigate whether informing about the nocebo effect using a short information sheet affects patients’ need for information about antidepressants.

**Methods:** A total of 97 patients taking recently prescribed antidepressants (≤4 months intake) were recruited over the internet and randomized to receiving either a one-page written information about the nocebo effect or a control text about the history of antidepressants. After experimental manipulation, informational needs about the side effects and mechanisms of antidepressants were assessed with 3 and 7 items on categorical and 5-point Likert scales. Group differences in informational needs were calculated with Chi-square tests and ANOVAs.

**Results:** Patients received antidepressants for depression (84.5%) and/or anxiety disorders (42.3%). Three participants (6.0%) of the nocebo group reported previous knowledge of the nocebo effect. After the experimental manipulation, participants in the nocebo group reported a reduced desire for receiving full side effect information [$X_{\left(4,97\right)}^{2}$ = 12.714, Cramer’s V = 0.362, *p* = 0.013] and agreed more frequently to the usefulness of withholding information about possible side effects [$X_{\left(4,97\right)}^{2}$ = 14.878, Cramer’s V = 0.392, *p* = 0.005]. Furthermore, they desired more information about the mechanisms of antidepressants (*F* = 6.373, *p* = 0.013, partial η^2^ = 0.063) and, specifically, non-pharmacological mechanisms, such as the role of positive expectations (*F* = 16.857, *p* < 0.001, partial η^2^ = 0.151).

**Conclusions:** Learning about the nocebo effect can alter patients’ informational needs toward desiring less information about the potential side effects of antidepressants and more information about general mechanisms, such as expectations. The beneficial effects of including nocebo information into contextualized informed consent should be studied clinically concerning more functional information-seeking behavior, which may ultimately lead to improved treatment outcomes, such as better adherence and reduced side effect burden.

## Introduction

In today’s Western healthcare systems, informed consent represents a fundamentally ethical and legal requirement for any medical intervention. It is considered an inherent part of evidence-based practice. However, by providing information about the medications’ potential side effects, practitioners may induce nocebo effects and cause harm (1, 2). Even reading the package leaflet of any given medication has been shown to increase side effect reporting (3, 4). Thus, informing a patient about a treatment provides a direct link to this treatments’ efficacy and tolerability. Ethically and clinically, this association has direct implications for informed consent procedures (5).

Nocebo effects may account for 38–100% of side effects reported in pharmacological trials, including serious adverse events (6). Particularly large placebo and nocebo effects have been documented in antidepressant treatment (7–10). A meta-analysis focusing on adverse event reporting showed that side effects specific to the drug emerge in the placebo groups of antidepressant medication trials, indicating that expectations are powerful enough to bias double-blind randomized trials (8). The role of expectations to predict the outcome of antidepressant treatment is prominent (11), but implications regarding the prevention of potential harm through negative expectations are rare. Clinical and experimental evidence suggests that nocebo-related side effects are caused by patients’ expectations (12–14), prior experiences, and conditioning processes (15, 16) as well as misattributions of pre-existing bodily symptoms (17) and social observation (18). Patients with depression might particularly be at risk due to frequent catastrophic thinking and, hence, are more prone to developing negative expectations (19, 20). In some patients, fear of side effects can be strong enough to motivate them to discontinue their antidepressant medication (21).

Antidepressant use is common, with an annual average of 1.52 billion daily doses prescribed in Germany (22). In the US, antidepressants are used by 13% of the country’s population, with a continuously increasing trend from 1999 to 2014 (23). Even though patient information procedures are essential to prescribing new drugs, their potential to optimize patients’ expectations remains untapped. Among other reasons, prescribing physicians might be unaware of the importance of contextual factors, such as the relevance of side effects information and patients’ expectations contributing to side effect burden (24). Common side effects associated with antidepressant treatment include headache, weight gain, dizziness, and dry mouth, as well as adverse effects of long-term antidepressant intake, such as emotional numbing (25).

Providing patients with comprehensive information about their medication is essential in light of patient autonomy. However, informing about side effects might also cause harm (26). To handle this ethical dilemma, promising approaches targeted to reduce expectation-induced side effects while still respecting patient autonomy and truthfulness have been suggested. Experimentally validated strategies include framing side effect information positively (4, 27), personalizing informed consent and educating about the medication’s mechanism of action (28), and explicitly informing about the nocebo effect itself (29). An important theoretical proposal suggests to contextualize the informed consent by providing medication information in a manner that is personalized to the patient’s characteristics, underlying disease, health status, and informational needs (30).

Contextualized informed consent might entail withholding information that may induce harm to patients. Being a theoretically discussed approach among experts, the patient’s view regarding this so-called authorized concealment remains unknown. Relevantly, very few individuals are aware of the nocebo effect and thus might not be able to express the need for respective medication information (29).

In this study, we will examine whether patients undergoing antidepressant treatment are open to receiving contextualized medication information, and in specific, what kind of information they wish or wish not to receive. Based on the assumption that knowledge about the nocebo effect might be prerequisite to contextualizing side effect information, we will inform one group of patients about nocebo effects and test whether this will influence patients’ informational needs. We assume that patients informed about the nocebo effect express a decreased need for detailed information about side effects and an increased need for information about the non-pharmacological mechanisms of side effect development in comparison with patients who were not informed about the nocebo effect.

## Methods and Procedures

### Participants

Participants for this study were recruited *via* four online depression forums, an advertisement on a local newspaper’s website, information sheets distributed in three different hospitals in the Hamburg metropolitan area, and four self-help groups. Inclusion criteria included a minimum age of 18, good knowledge of German, and having started a new antidepressant within the last 4 months.

### Study Design and Procedure

Ethics approval was obtained from the Psychotherapy Chamber Hamburg, Germany. The survey was assessed *via* an online link. On the first page, all participants were informed about study procedure and data storage. By checking a box on the website, informed consent was provided by all participants prior to study start. Participants were then asked to provide information on socio-demographic data, illness-related data, and their satisfaction with the received medication information. Then, participants were randomized to receiving either a short information about the nocebo effect or neutral information.

**Figure 1:**
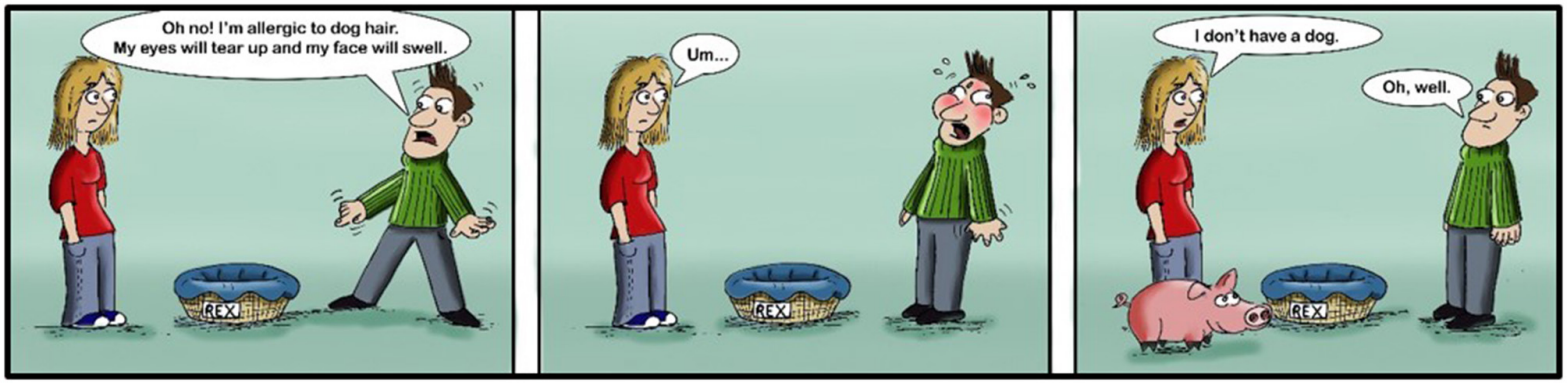
Illustration of nocebo effects in everyday life. ©Timm Kinitz.

The nocebo information group received a one-page text about the nocebo effect and its mechanisms; the control group received a text of the same length about the history of antidepressants. The nocebo information consisted of three main parts: a comprehensive explanation of the experienced nocebo effect, a distinction of pharmacological and non-pharmacological effects of a drug, and a description of expectations as one possible mechanism of the nocebo effect (31). Within the first paragraph, examples of expectations stemming from prior negative treatment experiences or from learning about the potential side effects from package inserts were given. It was further described that these negative expectations can lead to heightened side effects, that these symptoms are real and not “made up,” and that studies have shown that over half of the experienced side effects can be caused by expectations rather than by biomedical factors (17). The second paragraph detailed that medication side effects can be caused *via* two routes: through pharmacological mechanisms that are specific for the type of antidepressant medication and through non-pharmacological mechanisms, such as patients’ expectations. The third paragraph detailed that expectations can trigger biomedical changes within the body; furthermore, that expectations can lead to focused bodily attention, thereby making it likely for a person to attribute normal bodily sensations, such as benign headaches, as a side effect of a given medication. The text was followed by a three-panel comic illustrating the effect (see Figure 1). The control group text did not include information on the efficacy or mechanisms of action of antidepressant treatment. It described the clinical use of antidepressants since the 1950s and the different types of antidepressants that have since been prescribed to patients. A manipulation check was conducted using three single choice questions about the texts’ content. Participants were then asked about which medication information they would like to obtain and what degree of side effect disclosure they considered to be useful.

### Measures

#### Demographic and Medical Characteristics

The online survey assessed basic socio-demographic data, self-reported diagnosis, type of antidepressant medication, and utilized sources to receive information about their medication. Depression severity was assessed with the German short version of the Center for Epidemiological Studies—Depression (CES-D) scale (32, 33). Adherence was assessed *via* self-report using a prior validated single item (“How many pills have you actually taken during the last week?”) (34). Patients who took 80% or more pills were classified as adherent.

#### Satisfaction With Information About Medication

We developed five items to assess the satisfaction with the information about the antidepressant treatment: overall satisfaction, comprehensibility of the information, time and occasion to pose questions to the clinician, feeling sufficiently informed to take part in decision-making about the antidepressant treatment, feeling sufficiently informed to take part in decision-making about side effect treatments, and whether the information was delivered with kindness and respect. Each item is rated on a scale from 1 to 5 (1 “not at all,” 2 “rather not,” 3 “unsure,” 4 “rather satisfied,” 5 “very much satisfied”). We dichotomized the items for easier interpretation, with ratings of 4 and 5 grouped as “satisfied.” The satisfaction with the consultation time was assessed additionally.

We also used the Satisfaction with Information about Medicines Scale (SIMS) (35). The subscales satisfaction with information on “action and usage of medication” and “potential problems of medications” ranges from 0 to 9 and 0 to 8, with higher scores indicating a higher degree of satisfaction. A total score is calculated by adding up all items.

#### Outcome: Preferred Information Disclosure

##### Disclosure About Risks of Side Effects

We operationalized the preference for information by addressing two aspects. Patients were asked about their wish to be informed about the side effects and about the mechanisms of antidepressants.

The extent of informational needs about side effects was assessed with three items: (1) “Would you find it beneficial if your practitioner did *not* inform you about all possible side effects?,” rated as “very beneficial,” “beneficial, but only with my consent,” “undecided,” “not very beneficial,” or “not at all beneficial”; (2) “How thoroughly would you like your practitioner to inform you about possible side effects?,” rated from 1 “not at all” to 5 “very thoroughly”; and (3) “Which side effects would you like to be informed about?,” rated as “all side effects,” “only the most common ones,” “only the most severe ones,” “only the personally relevant ones,” or “none.”

##### Disclosure About Antidepressant Mechanisms

Informational needs about antidepressants’ mechanisms were measured with seven items on a scale from 1 “fully disagree” to 5 “fully agree.” Two items refer to the pharmacological mechanisms of antidepressants, whereas another five items refer to non-pharmacological mechanisms. In specific, patients were asked to indicate whether they would like their practitioner to inform them about the fact (1) “that antidepressants target messenger substances (neurotransmitters) in the brain,” (2) “that antidepressants have a pharmacological effect on the body *via* its biochemical pathways,” (3) “that my expectations about the treatment influence the effectiveness of the antidepressant,” (4) “that the antidepressant would be less effective if I was not convinced of its benefits,” (5) “that antidepressants have a non-pharmacological effect on the body (placebo effect) conveyed by hope for recovery or the attentive care of a physician,” (6) “that time itself can contribute to easing my suffering,” and (7) “that side effects can develop due to heightened attention to bodily sensations.”

#### Statistical Analyses

To compare the nocebo information group and the control group, Chi-square tests were conducted for categorical data, and *t*-tests for continuous variables. Welch *t*-tests were conducted if variances were unequal. Analyses were conducted using IBM SPSS 24. All tests were two-tailed with the alpha level set at 0.05.

## Results

### Sample Characteristics

Of 347 participants who started the online questionnaire, 102 participants completed the survey. Participants who could not identify their antidepressant medication (i.e., by checking a box within a comprehensive list of antidepressants) or who reported an intake time of more than 4 months were excluded. After completing the study, participants were excluded if completion time was two standard deviations above mean (*n* = 2), if they did not remember having received medication information from their prescribing physician (*n* = 2), or if they failed all questions of the manipulation check (*n* = 1). A total of 97 participants were included in the analyses, of which 49 and 48 were randomly allocated to the nocebo information group and to the control group, respectively.

Patients in both study groups were comparable with respect to socio-demographic characteristics (Table 1). When asked about their diagnosis, participants predominantly stated to receive antidepressants as treatment for depression (84.5%) and/or anxiety disorders (42.3%). More than 80% were still taking at least 80% of their medication; 10 participants have already discontinued antidepressant treatment. A majority received the medication information by their psychiatrist (54.6%) and used the internet (81.4%) or the package leaflet (73.2%) as an additional information source. Pre-existing knowledge of the nocebo effect was assessed in the nocebo information group using an open question; three participants (6%) could describe the effect correctly.

**Table 1:** Socio-demographic, medical characteristics, and satisfaction with medication information.

	**Nocebo information group (*n* = 49)** ***M* ±*SD* or % (*n*)**	**Control group** **(*n* = 48)** ***M* ±*SD* or % (*n*)**
Age	39.6 ± 10.0	38.6 ± 13.7
Female	59.2 (29)	54.2 (56)
Married/with partner	40.8 (20)	39.6 (19)
13 or more years of education	22.4 (11)	35.4 (17)
Employed	51.0 (25)	43.8 (21)
Diagnosis^a^		
Depression	85.7 (42)	83.3 (40)
Anxiety disorder	46.9 (23)	37.5 (18)
Bipolar disorder	6.1 (3)	10.4 (5)
Pain disorder	2.0 (1)	10.4 (5)
Obsessive compulsive disorder	2.0 (1)	4.2 (2)
Other	0.0 (0)	4.2 (2)
Type of antidepressants		
Citalopram	24.5 (12)	83.3 (13)
Venlafaxine	14.3 (7)	14.6 (7)
Escitalopram	14.3 (7)	4.2 (2)
Mirtazapine	8.2 (4)	10.4 (5)
Sertraline	8.2 (4)	10.4 (5)
Fluoxetine	6.1 (3)	6.3 (3)
Amitriptyline	0 (0)	8.4 (4)
Opipramol	4.1 (2)	2.1 (1)
Agomelatine	2.0 (1)	4.2 (2)
Other^b^	20.4 (10)	16.7 (8)
Depression severity (CES-D)	19.9 ± 9.6	17.9 ± 8.67
Adherent (80% or more pill intake)	86 (42)	81 (39)
Prescriber		
Psychiatrist	55.1 (27)	54.2 (26)
General practitioner	22.4 (11)	10.4 (5)
Practitioner in the clinic	16.3 (8)	14.6 (7)
Neurologist	6.1 (3)	18.8 (9)
Other	0 (0)	2.1 (1)
Satisfaction with Information (SIMS)		
Action and usage of medication^c^	6.1 ± 2.7	6.10 ± 2.6
Potential problems of medication^d^	3.7 ± 3.0	3.25 ± 2.9
Satisfaction with consultation duration		
Just right	49.0 (24)	68.8 (33)
Too short	42.9 (21)	31.3 (15)
Too long	8.2 (4)	0 (0)
Additional sources of information^a^		
Internet	81.6 (40)	81.3 (39)
Package leaflet	71.4 (35)	75.0 (36)
Patient brochures/ psychoeducation	24.5 (12)	12.5 (6)
Family/friends	10.2 (5)	12.5 (6)
Newspaper/TV	4.1 (2)	6.3 (3)
Self-help groups	2.0 (1)	4.2 (2)
Other	6.1 (3)	6.3 (3)
None	2.0 (1)	4.2 (2)

*M, mean; SD, standard deviation; CES-D, Center for Epidemiological Studies—Depression scale*.

a *Multiple responses allowed*.

b *Other antidepressants include drugs mentioned ≤2 times: Duloxetine, Clomipramine, Paroxetine, and Quetiapine*.

c *Range 0–9*.

d *Range 0–8*.

### Satisfaction With Information About Medication

**Figure 2:**
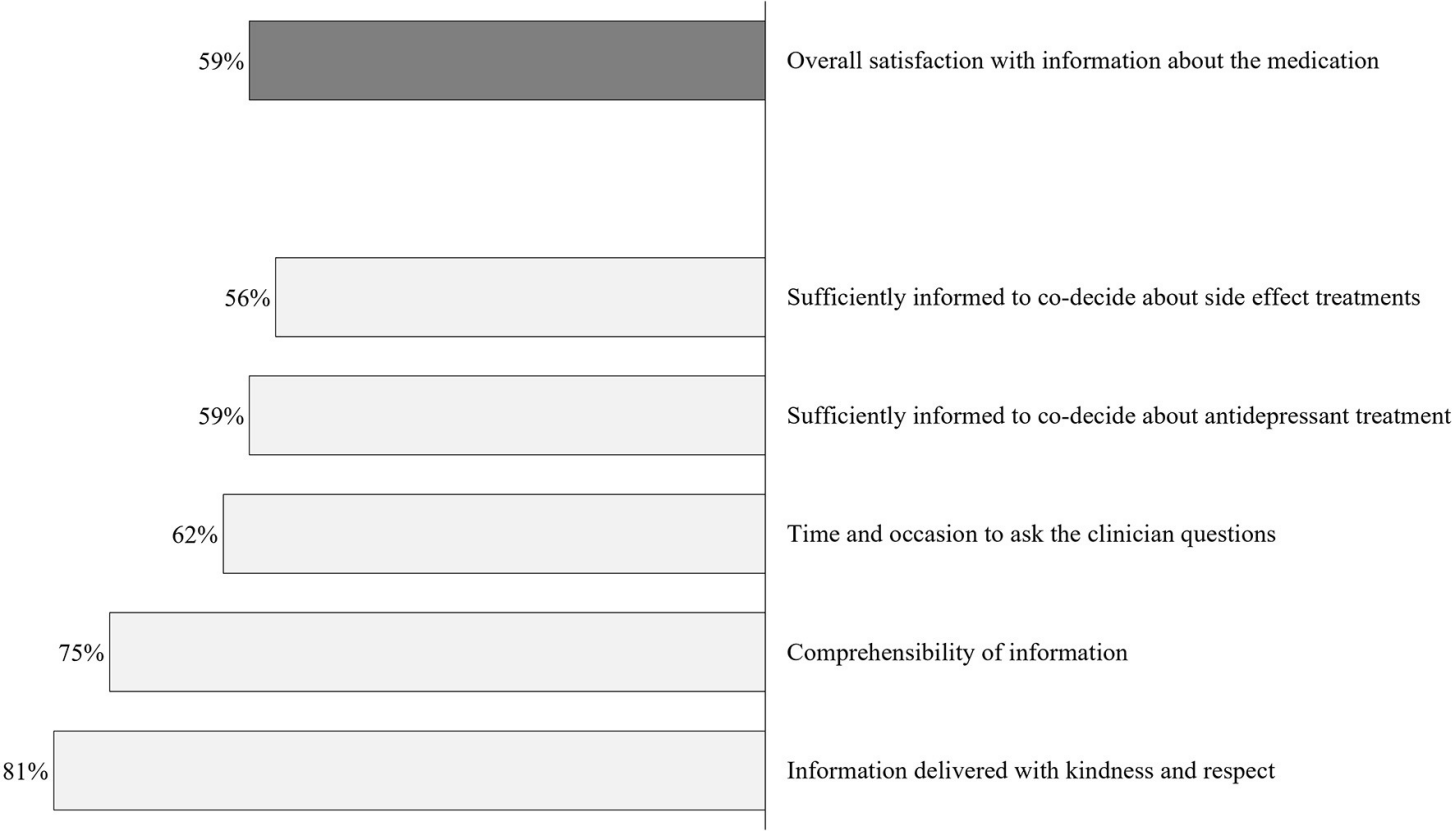
Satisfaction with information about medication at prescription. Percentages indicate the proportion of patients agreeing to each item (scores #3–5) (*N* = 97).

Figure 2 portrays the patients’ satisfaction with the medication information received from the prescribing physicians; 59% of patients were overall satisfied, yet 41% were not. Information was judged inadequate to participate in shared decision-making about side effect-related treatments by over 40% (44% not satisfied), and 41% felt inadequately informed to participate in shared decision-making about the antidepressant treatment (41% not satisfied). Considering consultation time, 58.8, 4.1, and 37.1% viewed the duration to be “just right,” “too long,” and “too short,” respectively.

Similarly, the SIMS indicated that patients were rather not satisfied with the obtained medication information. In comparison with the German norm population that consisted of *n* = 212 chronically ill patients in the primary care system (hypertension, musculoskeletal diseases, diabetes type 2, cardiac insufficiency), our patient sample reported lower satisfaction (SIMS scores) for the total information received [*M* = 9.59, *SD* = 4.87; *t*_(307)_ = 2.18, *p* = 0.006, Cohen’s *d* = 0.34], for the subscales action and usage of medication [*M* = 6.10, *SD* = 2.59; *t*_(307)_ = 2.19, *p* = 0.029, Cohen’s *d* = 0.27], and for the potential problems of medication [*M* = 3.48, *SD* = 2.91; *t*_(307)_ = 2.83, *p* = 0.006, Cohen’s *d* = 0.34].

### Informational Needs

#### Disclosure About Side Effects

**Figure 3:**
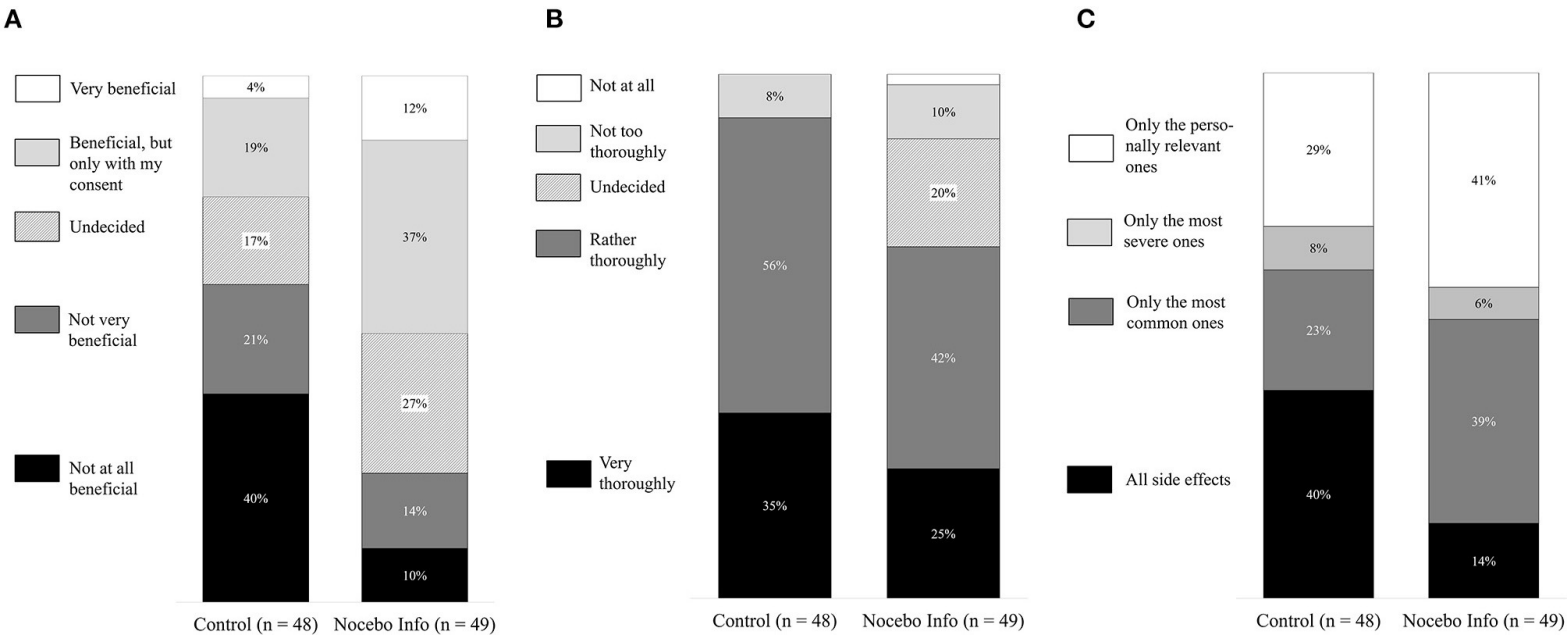
Preferred information disclosure about side effects. Black marked columns indicate the percentage of participants who wish to receive detailed information about the potential side effects. **(A)** Would you find it beneficial if your practitioner did not inform you about all possible side effects? **(B)** How thoroughly would you like your practitioner to inform you about possible side-effects? **(C)** Which side would you like to be informed about.

Figure 3 shows that the control group more strongly desired to be informed about all side effects. Chi-square tests revealed significant group differences regarding all three items on side effect disclosure. The groups differed considering the perceived benefits of not being informed about all possible side effects [$X_{\left(4\right)}^{2}$ = 14.88, *p* = 0.005, Cramer’s V = 0.39], considering the desire to be thoroughly informed about possible side effects [$X_{\left(4\right)}^{2}$ = 12.71, *p* = 0.013, Cramer’s V = 0.36], and considering the types of side effects they wish to be informed about [$X_{\left(3\right)}^{2}$ = 8.86, *p* = 0.031, Cramer’s V = 0.30].

#### Disclosure About the Antidepressant’s Modes of Actions

**Figure 4:**
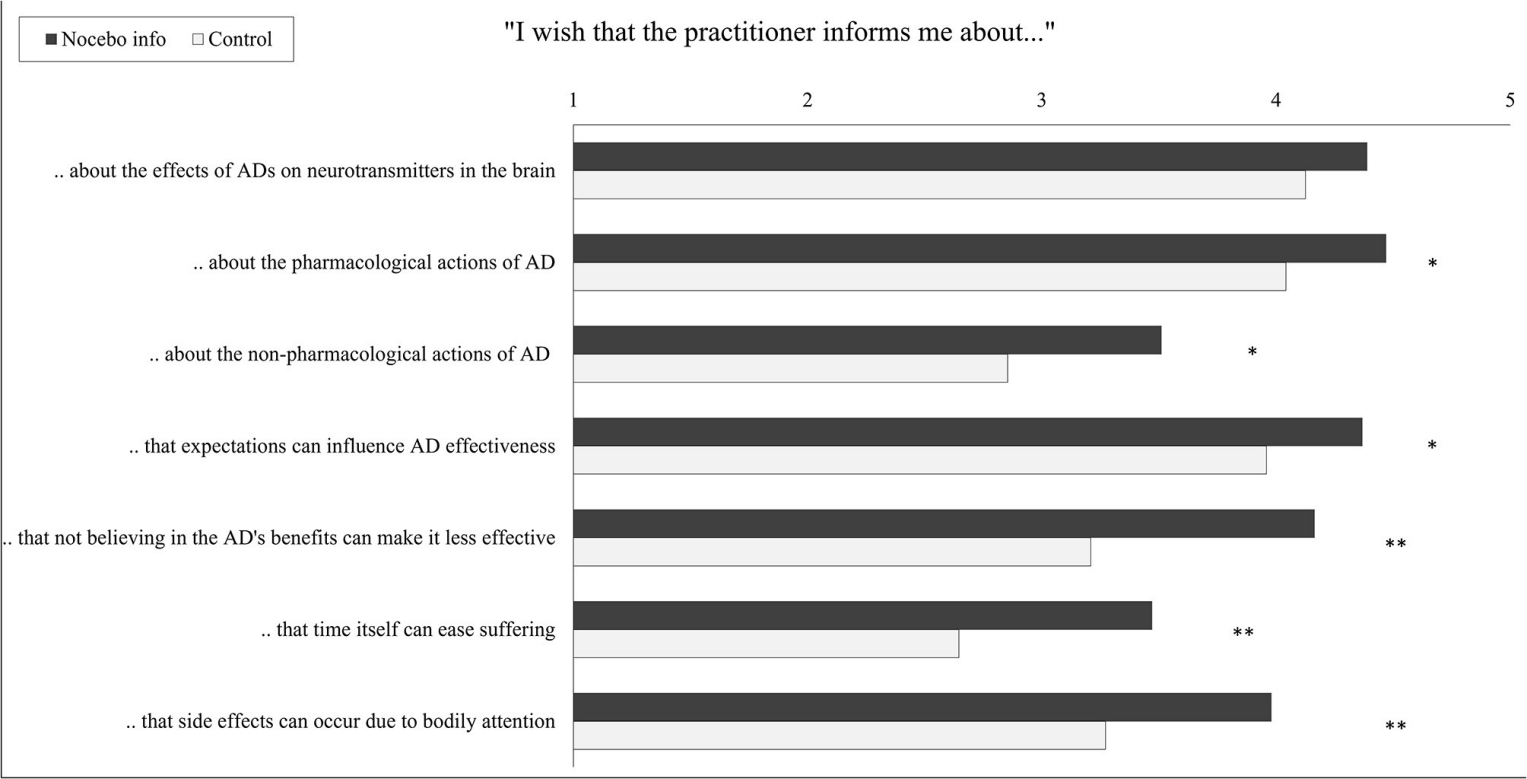
Wish for disclosure about modes of action of the antidepressant by the intervention group. Student’s *t*-tests for independent samples were conducted. Each scale ranges from 1 “do not agree at all” to 5 “fully agree”; bars indicate means. AD, antidepressant, ^*^p < 0.05, ^**^p < 0.01.

A multivariate analysis of variance (MANOVA) revealed a significant multivariate effect of the study group on informational needs about the mechanisms of antidepressants [Wilks’ λ = 0.75, *F*_(7, 89)_ = 3.64, *p* = 0.001, partial η^2^ = 0.25]. Except for information on “effects of antidepressants on neurotransmitters in the brain” (Figure 4), participants in the nocebo information group indicated an increased wish for information in all domains. *t*-Test for independent samples showed that the nocebo group desired more information about the pharmacological actions of antidepressants [*t*_(95)_ = 2.53, *p* = 0.013, Cohen’s *d* = 0.52], about the non-pharmacological actions of antidepressants [*t*_(95)_ = 2.52, *p* = 0.013, Cohen’s *d* = 0.52], on how expectations can influence the antidepressant’s effectiveness [*t*_(95)_ = 2.05, *p* = 0.043, Cohen’s *d* = 0.42], on how not believing in the antidepressant’s benefits can make it less effective [*t*_(88.07)_, *p* < 0.001, Cohen’s *d* = 0.83], on how time itself can ease suffering [*t*_(95)_ = 3.02, *p* = 0.003, Cohen’s *d* = 0.61], and about how side effect can develop due to heightened bodily attention [*t*_(95)_ = 2.98, *p* = 0.004, Cohen’s *d* = 0.61].

## Discussion

This study showed that patients, who have learned about the nocebo effect, are more open to contextualized information about their antidepressant medication. Patients with an indication for antidepressant treatment, in general, wish to be informed about the effects and potential side effects of their antidepressant medication. However, the group who has received information about the nocebo effect–in comparison with the group who did not receive that information–indicated that withholding the potential side effects and a less thorough disclosure of side effects would be beneficial. They also wished for a more personalized approach, i.e., 41% of participants wished to only be informed about personally relevant side effects (vs. 29% of participants in the control group).

Patients in the nocebo information group also reported an increased wish to be informed about the antidepressants’ mode of action, which includes pharmacological and non-pharmacological treatment mechanisms. This suggests that, once aware that psychological factors can contribute to side effects, participants were more receptive to information considering the medication’s non-pharmacological mechanisms. Especially for antidepressants, for which placebo effects determine up to 75% of the effectiveness (8–10, 36), nonspecific factors, such as expectations and positive beliefs, may influence treatment outcomes. Knowing about the nocebo effects provides the groundwork for learning more about non-pharmacological treatment mechanisms, which again, might positively affect treatment efficacy.

When asked about their overall satisfaction with the medication information they received at prescription, only 59% of the *n* = 97 participants indicated overall satisfaction. For most participants, the information was delivered with kindness and respect (81%) and was well comprehensible (75%). However, in view of “time and occasion for questions to the clinician,” fewer participants were satisfied (62%). Notably, many participants did not feel adequately informed to make decisions considering managing the potential side effects and their antidepressant treatment (satisfaction rates: 59 and 56%). In addition, the consultation time at prescription was evaluated as “too short” in 37% of all cases. These results can be interpreted as compatible with the current public health crisis of long-term antidepressant intake. About 14% of antidepressant users report an intake duration of 10 years (37), although there is no evidence for increased benefits for long-term intake (38, 39). On the other hand, many patients discontinue their medication without consulting their practitioner (40), which can result in heightened recurrence risk and burdening symptoms. Providing patients with more information at prescription might be an essential component of preventing abrupt discontinuation or the “better safe than sorry”-motivated long-term intake.

Up to 57% of patients experience nocebo effects from antidepressants (8, 41). Symptoms include dry mouth, fatigue, drowsiness, constipation, sexual problems, and vision/accommodation problems. Since all types of side effects seemed equally amendable to nocebo (8), it can be assumed that side effects from antidepressants in general might be influenced in terms of their incidence and intensity by patients’ negative expectations. Furthermore, 40% of the patients in the placebo groups of clinical trials discontinue antidepressant treatment because of intolerable side effects (8, 10). Taken together, nocebo effects from antidepressant treatment constitute a serious clinical problem affecting patients’ well-being as well as medication adherence. Clinical ways of tackling this problem, for example, through optimized informed consent procedures, thus seem promising not only for the benefit of the patient but also for the benefit of the healthcare systems struggling with costs from increased long-term antidepressant intake and self-directed discontinuation.

While there have been suggestions to inform patients about the nocebo effect (42), to the best of our knowledge, this study is the first to assess the patients’ wish for information after learning about the nocebo effect. A previous study showed that explaining the nocebo effect reduces symptomatic experiences in people reporting symptoms attributed to windfarm generated infrasound, supporting the potential positive impact of providing improved information about nocebo effects (43). Further strengths include the use of a control condition and the standardized presentation of the information, which could be easily implemented into medication package leaflets.

## Limitations

While the study’s online format minimized context effects and bias toward investigators, it does not provide opportunities for questions to a clinician, which could have enhanced the understanding of the nocebo effect. Clinically more valid routes to provide disclosure to patients about the nocebo effect include semi-structured individual consultations (31) or might be offered *via* shared clinical notes. Sharing clinical notes with patients *via* digitally accessible records, a practice that is becoming increasingly common in northern European countries and worldwide, might provide the opportunity to directly augment expectation effects (44). However, the ability to do this depends on the clinicians’ knowledge about the influence of expectations on treatment efficacy and tolerability (24). The claim to promote nocebo literacy by addressing expectation effects in clinical education has been recently raised in a consensus paper (45).

The sample size with 97 participating patients was rather small. Among other benefits, such as increased power, larger samples with an increased chance of including patient with diverse interests in expectation or nocebo effects might help to control for a potential responder bias, since interest in and experiences with these effects might influence patient’s informational needs. However, the study was promoted as a survey on experiences with antidepressants, which should have reduced a bias toward selective interests in the topic. All measures within this study were patient self-reports. Future studies should aim to include objective measures, such as pill counts as measures of adherence. Furthermore, the focus of this study was on patients taking antidepressants; thus, conclusions on other samples need to be drawn with caution. Since participants were not scheduled to receive a new prescription and had to answer hypothetically, future research should examine the information needs where the outcome actually determines the information of new medication given by a doctor.

Our study did not explore whether patients who are informed about the nocebo effect and prefer to receive fewer side effect information within their doctors’ consultation actually change their behavior in terms of decreased searching for negative information online and in their conversations with fellow patients. Thus, the potential of adapted informed consent procedures, such as authorized concealment, to really prevent nocebo effects should be investigated in further studies (46). Importantly, these future studies might also consider the potential risks and downsides of authorized concealment, such as increased anxiety or overlooking and downplaying of potentially serious adverse events due to their attribution to the nocebo effect (5). Moreover, physicians should not misinterpret a preference for lesser side effect information as a justification for providing less information about side effects in general. Conclusions from this scientific debate should always emphasize that patent autonomy, as one of the fundamental principles of informed consent, and in this case of authorized concealment, should remain intact.

## Conclusions

To our knowledge, our study is the first to investigate patients’ views of the potential contextualized informed consent procedures. In contrast to experts’ suggestions of withholding certain side effect information, most patients wish to receive information about all possible side effects. Only when patients have been informed about the nocebo effect, they agreed to receive adjusted medical information. Hence, patients should be informed about the underlying rationale of preventing nocebo effects before informed consent is contextualized. Future studies should investigate whether contextualized informed consent can optimize expectations as shown by Heisig et al. (28) and, furthermore, reduce side effect burden and improve the efficacy of medications.

Clinicians should be aware of the nocebo effect and provide information to the patient accordingly. This study shows that knowing about the nocebo effect can alter the need for information, which should be considered to achieve a truly informed consent and ensure patients’ autonomy.

## Data Availability Statement

The raw data supporting the conclusions of this article will be made available by the authors, without undue reservation.

## Ethics Statement

The studies involving human participants were reviewed and approved by Psychotherapy Chamber Hamburg, Germany. The patients/participants provided their written informed consent to participate in this study.

## Author Contributions

YN and MS-M initiated the study design. TK, EW, and MS-M conducted the study. TK, EW, YP, YN, and MS-M analyzed and interpreted the data. YN, YP, and TK drafted the manuscript. All authors made refinements and approved the final manuscript.

## Conflict of Interest

The authors declare that the research was conducted in the absence of any commercial or financial relationships that could be construed as a potential conflict of interest.
